# The Application of an Adaptive Genetic Algorithm Based on Collision Detection in Path Planning of Mobile Robots

**DOI:** 10.1155/2021/5536574

**Published:** 2021-05-07

**Authors:** Kun Hao, Jiale Zhao, Beibei Wang, Yonglei Liu, Chuanqi Wang

**Affiliations:** ^1^School of Computer and Information Engineering, Tianjin Chengjian University, Tianjin 300384, China; ^2^School of Control and Mechanical Engineering, Tianjin Chengjian University, Tianjin 300384, China; ^3^Tianjin Keyvia Electric Co., Ltd., Tianjin 300384, China

## Abstract

An adaptive genetic algorithm based on collision detection (AGACD) is proposed to solve the problems of the basic genetic algorithm in the field of path planning, such as low convergence path quality, many iterations required for convergence, and easily falling into the local optimal solution. First, this paper introduces the Delphi weight method to evaluate the weight of path length, path smoothness, and path safety in the fitness function, and a collision detection method is proposed to detect whether the planned path collides with obstacles. Then, the population initialization process is improved to reduce the program running time. After comprehensively considering the population diversity and the number of algorithm iterations, the traditional crossover operator and mutation operator are improved, and the adaptive crossover operator and adaptive mutation operator are proposed to avoid the local optimal solution. Finally, an optimization operator is proposed to improve the quality of convergent individuals through the second optimization of convergent individuals. The simulation results show that the adaptive genetic algorithm based on collision detection is not only suitable for simulation maps with various sizes and obstacle distributions but also has excellent performance, such as greatly reducing the running time of the algorithm program, and the adaptive genetic algorithm based on collision detection can effectively solve the problems of the basic genetic algorithm.

## 1. Introduction

With the development of the modern society, mobile robots have been widely used in shopping malls, factories, hospitals, and other public places [1, 2]. Mobile robots have already changed from the original simplification and mechanization to the direction of intelligence and humanization [3, 4]. Path planning technology is one of the key technologies to realize the intelligentization of mobile robots, and it is also a popular research topic in the field of mobile robots. Path planning means that the mobile robot searches for an optimal or suboptimal collision-free path from the starting point to the target point according to a certain performance index [5].

In the past few decades, path planning technology has been well developed. There are two kinds of path planning algorithms: traditional search algorithms and intelligent evolution algorithms. Traditional search algorithms include the Floyd algorithm [6], Dijkstra algorithm [7], artificial potential field method [8], and *A∗* algorithm [9]. Floyd algorithm [6] is easy to understand and simple to design, but Floyd algorithm is not suitable for a large amount of data because of its high time complexity. Dijkstra algorithm [7] is fast, but it cannot deal with the edge with negative weight. Therefore, the application of Dijkstra algorithm will be limited. The artificial potential field method [8] has a simple structure and has been widely used in real-time obstacle avoidance and smooth trajectory control, but the artificial potential field method has the problem of unreachable target near the obstacle and local minimum. *A*^*∗*^ algorithm [9] is suitable for simple maps. In complex maps, the program running time of *A*^*∗*^ algorithm will increase rapidly. Intelligent evolutionary algorithms include the neural network algorithm [10], immune clone algorithm [11], genetic algorithm [12], and ant colony algorithm [13]. Neural network algorithm [10] has strong fault tolerance and adaptability, but it needs a lot of computing resources and is not easy to use on mobile robots. Immune clonal algorithm [11] has fast convergence speed, but it is easy to fall into the local optimal solution. Moreover, the population diversity of immune clone algorithm is poor. Genetic algorithm [12] has the advantages of strong parallelism and robustness. However, genetic algorithm also has the problems of many iterations and easy to fall into the local optimum. Ant colony algorithm [13] adopts the pheromone positive feedback mechanism to make algorithm fast convergence. However, the ant colony algorithm has the deadlock problem. Deadlocks can kill large numbers of ants. In summary, the traditional search algorithm is suitable for small-scale maps with few obstacles. For large-scale and multiobstacle maps, the performance of traditional search algorithms is poor. The traditional search algorithm not only takes a long time to search but also easily falls into the local optimal solution. Compared with the traditional search algorithm, the intelligent evolutionary algorithm is applicable to both small-scale maps with few obstacles and large-scale maps with many obstacles. However, intelligent evolutionary algorithms are also prone to fall into local optimal solutions.

The genetic algorithm is an intelligent evolutionary algorithm. It is designed according to the theory of natural selection and the mechanism of genetic evolution. In [14], Holland et al. proposed the mathematical model of genetic algorithm. The model takes into account the nonlinearity of the complex interaction. They proved the universality of the model by applying it to economics, physiological psychology, game theory, and artificial intelligence. On the basis of this mathematical model, Holland et al. systematically introduced the principle of genetic algorithm and demonstrated the feasibility and scientificity of the genetic algorithm framework. In [15], Radu Emil Precup and Radu Codrut David introduced the technical progress and novelty of existing natural heuristic algorithms. They also demonstrated the optimization framework and basic principles of natural heuristic algorithms. On this basis, they systematically introduced the optimization algorithm of the fuzzy servo system and took it as an example. Genetic algorithm is widely used in the field of path planning because of its advantages, such as parallelism, strong robustness, and easy embedding into other algorithms. However, the basic genetic algorithm (BGA) also has some problems, such as low efficiency, low quality of convergent individuals, many iterations of convergence, and easily falling into the local optimal solution. To solve these problems, many scholars have performed much research on genetic algorithm. Viana et al. [16] improved the local search strategy in the traditional mutation operator and proposed a new multicrossover operator. This method can solve the premature problem of genetic algorithm. However, the performance of the algorithm is constrained by the fixed crossover probability and mutation probability. Choi et al. [17] proposed a hybrid algorithm based on a genetic algorithm and evolutionary strategy. The hybrid algorithm combines a genetic algorithm and evolutionary strategy. The genetic algorithm is used to approach the optimal solution, and the evolutionary strategy is used to solve the exact optimal solution. However, the hybrid algorithm is highly dependent on the algorithm parameters. When the parameter design is poor, the quality of the convergent individual obtained by this method is unstable. The nondominant sorting genetic algorithm (NSGA-III) has good diversity but poor convergence. Qingguo Liu et al. [18] added the *K*-means clustering algorithm on the basis of NSGA-III and proposed an improved genetic *K*-means clustering algorithm of NSGA-III. The improved algorithm is also known as NSGA-III-GKM. This algorithm can not only improve the convergence and diversity but also automatically provide the number and direction vectors of subspaces. However, the efficiency of this algorithm is low when solving MaOPs with complex constraints. Hao et al. [19] proposed a multipopulation migration genetic algorithm (MPMGA). In this algorithm, a large population is randomly divided into several small populations with the same population number, and the selection mechanism of the selection operator is replaced by the migration mechanism among the populations. The crossover operator and mutation operator are also improved. This algorithm can solve the problems of the standard genetic algorithm. However, the program running time of this algorithm is long, which limits its application in real life.

Through the above analysis, this paper presents an adaptive genetic algorithm based on collision detection (AGACD) for mobile robot path planning. This paper makes the following innovations and contributions: (1) this paper proposes a collision detection method to detect whether the planned path collides with the obstacle grid. The collision detection method is suitable for all static grid maps. Compared with other methods, the proposed method considers collision detection mathematically. Therefore, the applicability is very strong. (2) This paper introduces the Delphi weight method to evaluate the weight of path length, path smoothness, and path safety in the fitness function. Compared with the traditional fuzzy weight method, the Delphi weight method in this paper improves the practical application ability and flexibility of the algorithm. (3) Compared with other population initialization methods, the population initialization method proposed in this paper can greatly reduce the proportion of the initial population generation time to the total time, thus reducing the running time of the algorithm program. Therefore, the population initialization method proposed in this paper can enhance the real-time performance and practicability of the algorithm. (4) Compared with the traditional crossover operator and mutation operator, the adaptive crossover operator and adaptive mutation operator proposed in this paper can effectively reduce the numbers of invalid crossover and invalid mutation and improve the efficiency of the algorithm. (5) In simulation maps with different sizes and obstacle distributions, AGACD can always generate feasible and effective high-quality paths.

The remainder of this paper is organized as follows: in Section 2, we introduce the method of environment modeling. In Section 3, we introduce the collision detection algorithm suitable for grid maps in detail. In Section 4, we describe various aspects of AGACD in detail. In Section 5, we compare the performance of each algorithm and the path quality of each algorithm in two simulation environments of different sizes and different obstacle distributions. We also analyze the performance of each algorithm in detail. In Section 6, we summarize the paper. In Section 7, we summarize the shortcomings of AGACD. In light of these shortcomings, we discuss areas for future work.

## 2. Environment Modeling

**Figure 1:**
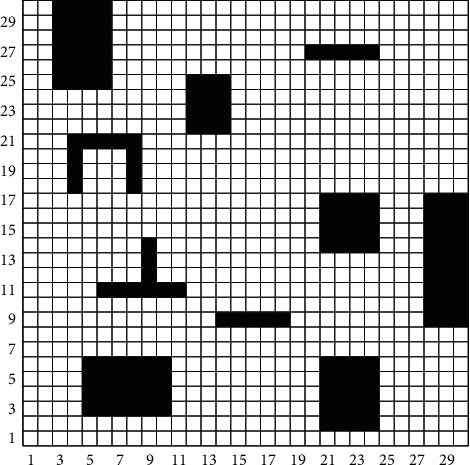
Grid map.

The common methods of environment modeling include the visual graph method, free-space method, and grid method. Because the grid method is easy to operate and understand, this paper uses the grid method to establish an environmental model. As shown in Figure 1, the whole two-dimensional workspace is divided into a 30*∗*30 grid map by the grid method. In the grid map, the serial numbers are 0, 1, 2, 3, 4, ..., 899 from left to right and from bottom to top. The white grid represents a feasible area, and the black grid represents an obstacle. The coordinates of the grid center point represent the grid coordinates. The correspondence between the grid serial number and grid coordinates is as follows:

(1) $$\begin{array}{c} \left\{\begin{array}{c} x=\text{mod}\left(p,N\right)+1,\\ y=\text{fix}\left(p/N\right)+1. \end{array}\right. \end{array}$$

(2) $$\begin{array}{c} p=\left(x-1\right)+\left(y-1\right)*N. \end{array}$$

Equations (1) and (2) represent the conversion between the grid serial number and the grid coordinates. In the two equations, *P* represents the grid serial number, *(x, y)* represents the coordinate point corresponding to the grid, *N* represents the grid number per row, mod represents the residual operation, and fix represents the integer operation.

To improve the security of the grid map, we need to preprocess it. Preprocessing includes expanding the obstacles and equating the mobile robot to the mass point. The expansion size is the sum of the radius of the mobile robot and the reserved safe distance.

## 3. Collision Detection Algorithm

Collision detection technology is the technology that detects whether the generated path collides with obstacles. Literature studies [20–22] propose several collision detection algorithms, but these algorithms are not applicable to the grid map shown in Figure 1. The literature [23, 24] has made outstanding contributions to the recognition and detection of obstacles under complex weather conditions. Therefore, on the premise of the accurate identification of obstacles, this paper proposes a collision detection algorithm that is suitable for grid maps. It is called the linear trial method.

**Figure 2:**
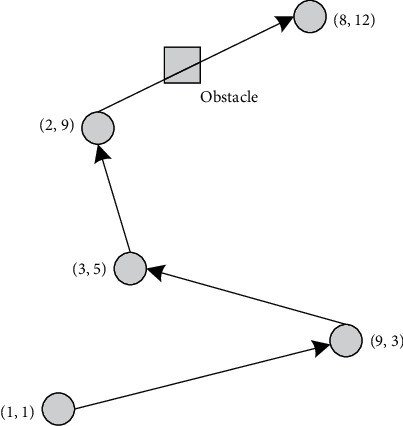
Schematic diagram of path collision detection.

Suppose a path consists of several path points. To detect whether a path collides with an obstacle grid, we only need to detect whether the connections of adjacent path points in the path collide with the obstacle grid. As shown in Figure 2, a path consists of five path points (1,1), (9,3), (3,5), (2,9), and (8,12). We only need to detect whether the (1,1), (9,3) path point connections, the (9,3), (3,5) path point connections, the (3,5), (2,9) path point connections, and the (2,9), (8,12) path point connections collide with the obstacle grid. If all adjacent path point connections do not collide with the obstacle grid, then the path is considered to have not collided with the obstacle grid. If any adjacent path point connections collide with the obstacle grid, then the path is considered to have collided with the obstacle grid. As shown in Figure 2, the (1,1), (9,3) path point connections, the (9,3), (3,5) path point connections, and the (3,5), (2,9) path point connections do not collide with the obstacle grid. However, the (2,9), (8,12) path point connections collide with the obstacle grid. Therefore, it can be concluded that the path collides with the obstacle grid.

Assuming that the coordinates of the two path points are *P*_*i*_(*x*_*i*_*,y*_*i*_) and P_*i+1*_(*x*_*i+1*_*,y*_*i+1*_), the linear equation of the line between the two points is

(3) $$\begin{array}{c} k=\left\{\begin{array}{c} \frac{\left(y_{i+1}-y_{i}\right)}{\left(x_{i+1}-x_{i}\right)},\;x_{i+1}\neq x_{i},\\ \infty ,\;x_{i+1}=x_{i}, \end{array}\right. \end{array}$$

(4) $$\begin{array}{c} b=y_{i}-k*x_{i},\;k\neq \infty , \end{array}$$

(5) $$\begin{array}{c} \left\{\begin{array}{c} y=k*x+b,\;k\neq \infty \\ x=x_{i},\;k=\infty . \end{array}\right. \end{array}$$

Equations (3)–(5) are the linear equation computations.

Let dy *=* *|y*_*i+1*_ *-* *y*_*i*_*|* and dx *=* *|x*_*i+1*_ *-* *x*_*i*_*|*. If dy is greater than d*x*, the *y* coordinate is used for detection; otherwise, the *x* coordinate is used for detection.

**Figure 3:**
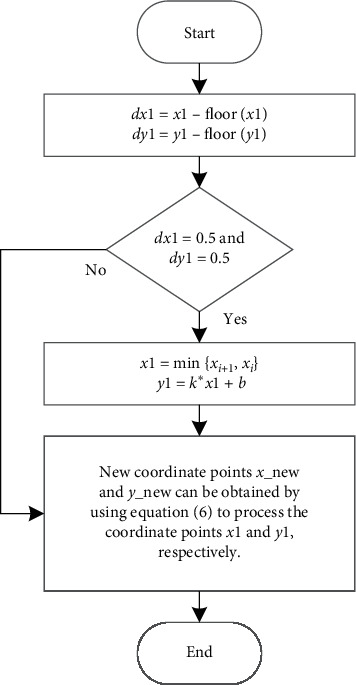
Data processing flowchart.

Assuming that dx is greater than dy, that is, the *x* coordinate is adopted for detection, then the following procedure can be followed:

1. Let *x1* *=* *min{x*_*i+1*_, *x*_*i*_*}, x2* *=* *max{x*_*i+1*_, *x*_*i*_*}.*
2. If *x1* *<* *x2*, *x1* *=* *x1* *+* *0.1*; proceed to Step (3). Otherwise, the process ends, and we determine that the connection between the two path points does not go through the obstacle grid.
3. We need to use equation (5) to solve *y*1 corresponding to *x*1, and then we can obtain the corresponding coordinate (*x*1, *y*1). Since the environment model established by the grid method can only identify whether the grid corresponding to the inherent coordinate points is an obstacle grid, the data processing flowchart shown in Figure 3 is used to process *x*1 and *y*1, and *x*_new and *y*_new can be obtained. (*x*_new, *y*_new) is a new coordinate that can be recognized. Go to Step (4).
4. We use equation (2) to convert the coordinates (*x*_new, *y*_new) into the grid number *p*_new and determine whether *p*_new is an obstacle grid. If *p*_new is not an obstacle grid, then proceed to Step (2). If *p*_new is the obstacle grid, the process ends, and we determine that the connection between the two path points goes through the obstacle grid.

Equation (6) is as follows:

(6) $$\begin{array}{c} \text{new}=\left\{\begin{array}{c} \text{floor}\left(u\right),\;u-\text{floor}\left(u\right)\leq 0.5,\\ \text{floor}\left(u\right)+1\text{,}\;u-\text{floor}\left(u\right)>\text{0}.5. \end{array}\right. \end{array}$$

In equation (6), *u* is the data to be processed, new is the processed data, and floor() is a downward rounding function.

The above procedure uses *x*-coordinates for detection. The process of detection using *y*-coordinates is the same as the process of detection using *x*-coordinates. We just need to replace *x* and *y* with each other.

From the detection process, we can see that the essence of the linear trial method is to gradually probe from the starting coordinate point to the target coordinate point by increasing the coordinate value by 0.1 each time. Since the environment model established by the grid method can only identify whether the grid corresponding to the inherent coordinate points is an obstacle grid, the tested coordinate values need to be processed. We need to check whether the grid corresponding to the processed coordinate value is the obstacle grid to judge whether the connecting line between the two path points passes through an obstacle grid.

## 4. Algorithm Design

### 4.1. Algorithm Framework

**Figure 4:**
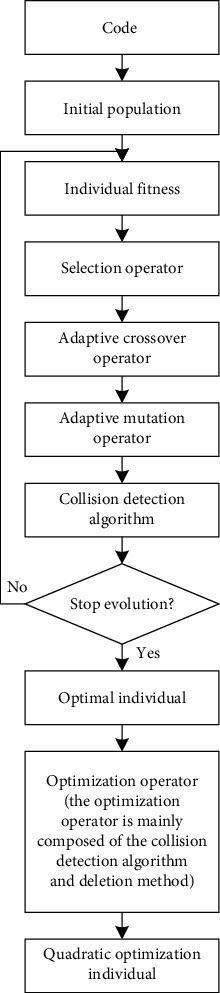
Frame diagram of the adaptive genetic algorithm based on collision detection.

Based on the basic genetic algorithm, an adaptive genetic algorithm based on collision detection is proposed in this paper. In Figure 4, the initial population with several individuals is generated randomly. Compared with the traditional population initialization method, this paper adopts prior knowledge and random disturbance method. This population initialization method can effectively reduce the running time of the algorithm program and ensure the diversity of the initial population. Because of the new method of population initialization, these path individuals are collision-free paths. Thus, they do not require collision detection. Then, the fitness function is used to calculate the fitness of each individual in the population. In terms of fitness, this paper uses the Delphi weight method to evaluate the weight of each optimization objective to obtain a set of weights close to the actual situation. This fitness calculation method enhances the application ability of the algorithm in real life. Next, based on the fitness of each individual in the population, individuals are selected, crossed, and mutated. Compared with the traditional selection operator, this paper adopts the method of combining the roulette selection strategy with the elite selection strategy. On the one hand, this selection strategy can accelerate the convergence speed of the algorithm. On the other hand, this selection strategy can prevent the algorithm from data fallback phenomena. In terms of the crossover operator and mutation operator, an adaptive crossover operator and adaptive mutation operator are proposed in this paper. These two adaptive operators can not only protect high-quality individuals to speed up the convergence rate of the algorithm but also enhance the evolutionary potential of low-quality individuals to increase the searching ability of the algorithm. Since both crossover and mutation operations generate infeasible path individuals, it is necessary to use a collision detection algorithm to remove these infeasible path individuals and then proceed to the next operation. We obtain the best individuals after the end of evolution. In this paper, the point deletion method and collision detection algorithm are fused to design an optimization operator. The optimization operator is used to optimize the optimal individual, and the quadratic optimization individual is obtained.

### 4.2. Coding Mode

The common encoding methods include binary encoding, floating point encoding, and real encoding. The disadvantages of binary coding are the large amount of data, low computing efficiency, and high memory overhead. In addition, binary coding is not easy to operate. Floating point encoding is suitable for continuous environments, but the encoding accuracy is low in discrete environments. Real number coding is easy to operate and easy to understand. Real number coding has high efficiency and low memory overhead. Therefore, real number coding is adopted in this paper.

### 4.3. Initial Population

Hao et al. [19] pointed out that, with the increase in the map scale, the proportion of the time taken to generate the initial population relative to the whole algorithm program time will gradually increase. In a 50 *∗* 50 grid map, the time taken to generate the initial population accounts for 81.32% of the time taken by the whole algorithm. Therefore, the time to generate the initial population will directly affect the time of the whole algorithm program. In this paper, we will reduce the time and improve the quality of the initial population by improving the method of initial population generation.

In this paper, prior knowledge and random perturbation are used to initialize the population. Assuming that the list table is empty and the starting node is added to the list table, the specific steps to generate an individual are as follows:

1. In the grid map, we choose the end point as the target point with a 50% probability and any free grid as the target point with the remaining 50% probability. Go to Step (2).
2. We need to calculate the Euclidean distance of the neighbor grid around the current grid node to the target grid. If the neighbor grid is an obstacle grid, the Euclidean distance between the neighbor grid and the target grid is denoted as infinite. Go to Step (3).
3. Through Euclidean distance, we select the nearest neighbor grid to the target grid as the current grid node and add it to the list table. If the neighbor grid is an end point, the individual generation ends, and the grid serial numbers in the list table are the generated path individual. If the neighbor grid is not an end point, go to Step (1).

Through the above steps, we can generate an individual. If the number of initial populations is *m*, we only need to execute the above steps *m* times.

### 4.4. Fitness Function

Fitness is usually used to evaluate the quality of an individual. In this paper, path length, path smoothness, and path safety are considered comprehensively. Haber et al. and Guerra et al. [25, 26] introduced two kinds of multiobjective optimization strategies and methods and achieved very good optimization results. On the basis of these two literature studies, we transform the multiobjective optimization problem into a single-objective optimization problem by the form of a weighted sum and use the Delphi weight method to evaluate the weight of multiple objectives. The total fitness function in this paper is defined as follows:

(7) $$\begin{array}{c} \text{fitness}=w1*f1+w2*f2+w3*f3. \end{array}$$

In equation (7), *f1* represents the fitness function of path length, *f2* represents the fitness function of path smoothness, and *f3* represents the fitness function of path safety. *w1*, *w2*, and *w3* are the weights of the three fitnesses, and the sum is 1.

*f1*, *f2*, and *f3* are defined as follows:

(8) $$\begin{array}{c} \left\{\begin{array}{c} f1=\frac{1}{\text{path}},\\ f2=\frac{1}{\text{smoothness}}\text{,}\\ f3=\frac{1}{\text{safety}}. \end{array}\right. \end{array}$$

In equation (8), path represents the path length, smoothness represents the path smoothness, and safety represents the path safety.

The path length is generally calculated by Euclidean distance. If a path is composed of *n* path points, the coordinate of the *i*th path point is *p*_i_(*x*_i_, *y*_i_), and the coordinate of the *i *+ 1 path point is *p*_*i*+1_(*x*_*i*+1_, *y*_*i*+1_), then the path length can be expressed as

(9) $$\begin{array}{c} \text{path}=\sum_{i=1}^{n-1}\sqrt{{\left(x_{i+1}-x_{i}\right)}^{2}+{\left(y_{i+1}-y_{i}\right)}^{2}}. \end{array}$$

Path smoothness refers to the cumulative sum of rotation angles of a mobile robot in a path. Path smoothness is generally used to measure the smoothness of the entire path. A large path smoothness value indicates that the path is not smooth. The path smoothness is usually calculated by the rotation angle. Suppose a path is composed of *n* path points. The coordinate of the *i *− 1 path point is *p*_*i*−1_(*x*_*i*−1_, *y*_*i*−1_), the coordinate of the *i*th path point is *p*_*i*_(*x*_*i*_, *y*_*i*_), and the coordinate of the *i *+ 1 path point is *p*_*i*+1_(*x*_*i*+1_, *y*_*i*+1_). Three continuous path points can form two path segments *p*_*i*−1_*p*_*i*_ and *p*_*i*_*p*_*i*+1_. Assuming *θ*_i_ is the rotation angle between *p*_*i*−1_*p*_*i*_ and *p*_*i*_*p*_*i*+1_, the smoothness of the path can be expressed as

(10) $$\begin{array}{c} \text{smoothness}=\sum_{i=2}^{n-1}\theta_{i}. \end{array}$$

The calculation equation of *θ*_*i*_ is as follows:

(11) $$\begin{array}{c} k1=\left\{\begin{array}{c} \frac{\left(y_{i}-y_{i-1}\right)}{\left(x_{i}-x_{i-1}\right)},\;x_{i}\neq x_{i-1},\\ \infty ,\;x_{i}=x_{i-1}, \end{array}\right. \end{array}$$

(12) $$\begin{array}{c} k2=\left\{\begin{array}{c} \frac{\left(y_{i+1}-y_{i}\right)}{\left(x_{i+1}-x_{i}\right)},\;x_{i+1}\neq x_{i},\\ \infty ,\;x_{i+1}=x_{i}, \end{array}\right. \end{array}$$

(13) $$\begin{array}{c} \theta_{i}=\left\{\begin{array}{c} 0,\;k1=\infty ,\;k2=\infty ,\\ 0,\;k1=k2k1,\;k2\neq \infty ,\\ \pi -\alpha_{i},\;\text{else}, \end{array}\right. \end{array}$$

(14) $$\begin{array}{c} \left\{\begin{array}{c} a1={\left(x_{i-1}-x_{i}\right)}^{2}+{\left(y_{i-1}-y_{i}\right)}^{2},\\ b1={\left(x_{i+1}-x_{i}\right)}^{2}+{\left(y_{i+1}-y_{i}\right)}^{2},\\ c1={\left(x_{i-1}-x_{i+1}\right)}^{2}+{\left(y_{i-1}-y_{i+1}\right)}^{2}, \end{array}\right. \end{array}$$

(15) $$\begin{array}{c} \left\{\begin{array}{c} a=\sqrt{a1},\\ b=\sqrt{b1},\\ c=\sqrt{c1}, \end{array}\right. \end{array}$$

(16) $$\begin{array}{c} d_{i}=\frac{\left(a1+b1-c1\right)}{\left(2*a*b\right)}, \end{array}$$

(17) $$\begin{array}{c} \alpha_{i}=\;\cos^{-1}d_{i}. \end{array}$$

Equations (11) and (12) calculate the slopes of *p*_*i*−1_*p*_*i*_ and *p*_*i*_*p*_*i*+1_ in the two path segments, respectively. Equation (13) discusses the value of *θ*_*i*_ according to the relationship between the slope of two path segments. In equation (13), *α*_*i*_ represents the included angle between the two path segments *p*_*i*−*1*_*p*_*i*_ and *p*_*i*_*p*_*i*+1_. Equations (14)–(17) are the calculation process of *α*_*i*_. In equation (17), cos^−1^ represents the arccosine function.

Path safety is usually used to measure the safety of a path. A large path safety degree indicates that the path is not safe. The path safety degree is generally calculated by the sum of the safety penalty degree obtained by each path point in a path. Assuming that a path is composed of *n* path points and the coordinate of the *i*th path point is *p*_*i*_(*x*_*i*_, *y*_*i*_), the path safety degree can be expressed as

(18) $$\begin{array}{c} \text{satefy}=1+\sum_{i=1}^{n}S_{i}, \end{array}$$

(19) $$\begin{array}{c} S_{i}=\sum_{j=1}^{8}\text{punishment}\_\omega_{j}, \end{array}$$

(20) $$\begin{array}{c} \text{punishment\_}\omega_{\text{j}}=\left\{\begin{array}{c} \text{0}.1\;\text{if }\omega_{j}\text{ is an obstacle grid and does not provide a security penalty for other path points,}\\ \text{0}\;\text{if }\omega_{j}\text{ is not an obstacle grid,}\\ \text{0}\;\text{if }\omega_{j}\text{ is an obstacle grid but has provided a security penalty for other path points.} \end{array}\right. \end{array}$$

In equation (18), *S*_*i*_ represents the degree of safety penalty obtained by the *i*th path point. In equation (19), punishment_*ω*_*j*_ represents the degree of security penalty provided by the *j*th grid around the *i*th path point. *ω*_*j*_ represents the *j*th grid around the *i*th path point. In equation (20), if *ω*_*j*_ is an obstacle grid and does not provide the safety penalty degree for other path points, *ω*_*j*_ will provide a 0.1 safety penalty degree for the *i*th path point. If *ω*_*j*_ is not an obstacle grid, *ω*_*j*_ does not provide a safety penalty to the *i*th path point (i.e., the safety penalty provided by *ω*_*j*_ is 0). If *ω*_*j*_ is an obstacle grid but already provides a safety penalty for other path points, *ω*_*j*_ will not provide a safety penalty for the *i*th path point.

In the calculation of the total fitness, determining the weight coefficients is a key issue. In this paper, the Delphi weight method is used to evaluate each weight, and a set of good weight coefficients is obtained.

First, we need to specify the order of importance of indicators according to actual needs. In this paper, the order of the importance of the three indicators is path length, path smoothness, and path security. Thus, we obtain *w1* ≥ *w2* ≥ *w3*.

The importance ratio is defined as follows:

(21) $$\begin{array}{c} I_{k}=\frac{wk}{w\left(k+1\right)}. \end{array}$$

Hence, we have *I*_*k*_*>=1*.

The importance ratio table constructed in this paper is shown in Table 1.

According to the ratio *I*_k_, *w*3 is calculated as follows:

(22) $$\begin{array}{c} \overset{2}{\underset{k=1}{\sum }}\left(\overset{2}{\underset{i=k}{\prod }}I_{k}\right)=\frac{\sum_{k=1}^{2}wk}{w3}, \end{array}$$

(23) $$\begin{array}{c} 1+\overset{2}{\underset{k=1}{\sum }}\left(\overset{2}{\underset{i=k}{\prod }}I_{k}\right)=w3^{-1}, \end{array}$$

(24) $$\begin{array}{c} w3={\left(1+\sum_{k=1}^{2}\left(\prod_{i=k}^{2}I_{k}\right)\right)}^{-1}. \end{array}$$

This article specifies *I*_*1*_ *=* 1.2 and *I*_*2*_ *=* 1.4, that is, path length is slightly more important than path smoothness, and path smoothness is more important than path security.

Then, we can calculate *w*1, *w*2, and *w*3:

(25) $$\begin{array}{c} \left\{\begin{array}{c} w1=0.4118,\\ w2=0.3431,\\ w3=0.2451, \end{array}\right. \end{array}$$

that is, the weight coefficients of path length, path smoothness, and path security are 0.4118, 0.3431, and 0.2451, respectively.

### 4.5. Selection Operator

Common selection strategies include roulette selection, elite selection, tournament selection, and truncation selection. However, there are a variety of problems with these selection strategies. For example, roulette selection calculates the probability of each individual appearing in offspring based on the fitness value of the individual and randomly selects individuals according to this probability. The intention of roulette selection is that the greater the fitness, the greater the probability that an individual will be selected. However, roulette selection includes the possibility that the best individual is not selected. Elite selection will lead the genetic algorithm to converge to a local optimum. Truncation selection is a standard method in animal and plant breeding, but it is not suitable for path planning. Tournament selection lacks random noise, that is, the poor individual will never survive, and the good individual will always win the tournament. This will lead to a rapid decrease in the population diversity of the genetic algorithm.

Based on the above problems, this paper improves the roulette selection strategy and integrates roulette selection with elite selection. The roulette selection strategy can enhance the diversity of the population and enlarge the search range of the solution space. Roulette selection can also solve the defect that elite selection converges to a local optimal solution. The elite selection strategy is beneficial to the preservation of the best individuals in the population. Elite selection solves the problem that the best individuals may not be selected in roulette selection. The two strategies can complement each other so that the algorithm can quickly converge to a high-quality solution.

### 4.6. Adaptive Crossover Operator

The crossover operator is mainly used in the global search of the algorithm. Common crossover operators include single-point crossover, multipoint crossover, and uniform crossover. Because the single-point crossover operation is simple, efficient, and convenient, this paper adopts the single-point crossover method.

Regarding cross-probability, the traditional cross-probability is a fixed constant value. Obviously, the traditional crossover probability cannot meet the needs of different individuals for different crossover probabilities. In fact, in the early stage of evolution, the algorithm needs a strong global search ability; at this time, the algorithm needs a large crossover probability. In the later stage of evolution, the algorithm needs a weak global search ability; at this time, the algorithm needs a small crossover probability. For different individuals in the population, the high-quality individuals need to be protected, so the high-quality individuals need to be given a small probability of crossover. Inferior individuals need to evolve, so inferior individuals need to be given a greater probability of crossover. Liu Jianwen et al. [27] proposed an adaptive crossover probability based on the individual similarity. However, this adaptive crossover probability only considers the relationship among individuals in the population and does not consider the relationship between individuals and the number of iterations. Therefore, a new adaptive crossover probability is proposed in this paper. The new adaptive crossover probability not only considers the relationship among individuals in the population but also considers the relationship between individuals and the number of iterations.

The new adaptive crossover probability equation is as follows:

(26) $$\begin{array}{c} \text{pc\_temp}=\left\{\begin{array}{c} \text{pc\_low,}\;f\text{\_max}=f\text{\_avg,}\\ \left.\frac{\text{pc\_high}*\left(f\_\max-f\right)}{f\_\max-f\_\text{avg}}\right)\text{,}\;f\geq f\_\text{avg and }f\_\max\neq f\_\text{avg},\\ \text{pc\_high,}\;f<f\_\text{avg and }f\_\max\neq f\_\text{avg,} \end{array}\right. \end{array}$$

(27) $$\begin{array}{c} \text{pc}=\text{pc\_temp}*e^{-t/T}. \end{array}$$

In equation (26), pc_temp is a parameter in equation (27). *f* is the larger fitness value in the corresponding fitness value of the two selected individuals. *f*_max is the maximum fitness value in the whole population. *f*_avg is the average fitness of the entire population. pc_high is a fixed constant. It is greater than pc_low and is between 0 and 1. pc_low is a fixed constant. It is less than pc_high, and it is between 0 and 1. When *f*_max is equal to *f*_avg, it means that the population has converged or is close to converging. pc_temp will be given a very small value pc_low. When *f*_max is not equal to *f*_avg and *f* is less than f_avg, it means that the individual is inferior and requires more evolution. pc_temp is given a value pc_high. When *f*_max is not equal to *f*_avg and *f* is greater than or equal to *f*_avg, it means that the individual is better. pc_temp is assigned a value according to a specific probability equation. In equation (27), *e* is the base of the natural logarithm function. *T* is the total number of iterations, and *t* is the current number of iterations.

### 4.7. Adaptive Mutation Operator

The mutation operator is mainly used in the local search of the algorithm. Common mutation methods include single-gene point mutation and multigene point mutation. Because the location and number of mutation points are uncertain, the operation of the multigene point mutation is complicated. Moreover, multigene point mutation also easily affects the efficiency of the algorithm. Therefore, single-gene point mutation is selected in this paper.

Similar to the traditional crossover probability, the traditional mutation probability is also a fixed constant value. Obviously, the traditional mutation probability cannot meet the needs of different individuals for different mutation probabilities. In fact, in the early stage of evolution, the algorithm needs a weak local search ability; at this time, the algorithm needs a small mutation probability. In the later stage of evolution, the algorithm needs a strong local search ability; at this time, the algorithm needs a larger mutation probability. For different individuals in the population, high-quality individuals need to be protected, so high-quality individuals need to be given a small probability of mutation. Inferior individuals need to evolve, so inferior individuals need to be given a greater probability of mutation. Based on the above analysis, an adaptive mutation probability is proposed in this paper. The adaptive mutation probability not only considers the relationship among individuals in the population but also considers the relationship between individuals and the number of iterations.

The adaptive mutation probability equation is as follows:

(28) $$\begin{array}{c} \text{pm\_temp}=\left\{\begin{array}{c} \text{pm\_high,}\;f\text{\_max}=f\text{\_avg,}\\ \frac{\text{pm\_low}\left(f\text{\_max}-\text{f}\right)}{\left(f\text{\_max}-\text{f\_avg}\right)}\text{,}\;f\text{\_avg and }f\_\text{max}\neq f\text{\_avg,}\\ \text{pm\_high,}\;f<f\text{\_avg and }f\text{\_max}\neq \text{f\_avg,} \end{array}\right. \end{array}$$

(29) $$\begin{array}{c} \text{pm}=\text{pm}\_\text{temp}*e^{t/T}. \end{array}$$

In equation (28), pm_temp is a parameter in equation (29). *f* is the fitness value of the selected individual. *f*_max is the maximum fitness value in the whole population. *f*_avg is the average fitness of the entire population. pm_high is a fixed constant. It is greater than pm_low and is between 0 and 1. pm_low is a fixed constant. It is less than pm_high and is between 0 and 1. When *f*_max is equal to *f*_avg, it means that the population has converged or is close to converging. pm_temp will be given a value pm_high to try to break the current optimal solution. When *f*_max is not equal to *f*_avg and *f* is less than *f*_avg, it means that the individual is inferior and requires more evolution. pm_temp is given a value pm_high. When *f*_max is not equal to *f*_avg and *f* is greater than or equal to *f*_avg, it means that the individual is better. pm_temp is assigned a value according to a specific probability equation. In equation (29), *e* is the base of the natural logarithm function. *T* is the total number of iterations, and *t* is the current number of iterations.

### 4.8. Optimization Operator

The optimization operator is an operator that performs a second optimization on a high-quality individual which has converged. The basic idea of the optimization operator is to simplify the path by removing the redundant points in the path. For example, a path consists of five path points A, B, C, D, and E. These five path points are in turn the adjacent path points. Thus, they can form four path segments AB, BC, CD, and DE. Suppose none of these four path segments go through obstacles. We try to remove path point B, directly connect path point A and path point C, and determine whether path segment AC passes through obstacles. If path segment AC does not pass through obstacles, path point B is the redundant path point. Then, we delete path point B from the path. The collision detection algorithm provided in Section 3 is used to determine whether a path segment passes through obstacles. After deleting path point B from the path, there are still four path points A, C, D, and E in this path. We try to remove path point C; then, we directly connect path point A and path point D and determine whether path segment AD passes through obstacles. If path segment AD passes through obstacles, then path point C is not a redundant path point. We try to remove path point *D*; then, we directly connect path point C and path point E and determine whether path segment CE passes through obstacles. If path segment CE does not pass through obstacles, path point D is the redundant path point. Then, we delete path point D from the path. The process of optimizing the path ends.

Thus, a collision-free path with five path points can be optimized to a collision-free path with three path points.

## 5. Simulation Results and Analysis

### 5.1. Simulation Environment

To verify the performance of the adaptive genetic algorithm based on collision detection in the field of path planning, this paper compares and analyzes the path generation, optimal individual fitness, and algorithm running time of the adaptive genetic algorithm based on collision detection (AGACD) and the basic genetic algorithm (BGA) under the 20 *∗* 20 grid map environment. In the environment of a 50 *∗* 50 grid map, performance measures such as path generation, optimal individual fitness, and running time are compared and analyzed for the adaptive genetic algorithm based on collision detection, basic genetic algorithm, and multipopulation migration genetic algorithm (MPMGA) proposed in [19].

The hardware and software configuration of the simulation experiment are shown in Table 2.

### 5.2. Simulation Experiment Based on the 20 *∗* 20 Grid Map

**Figure 5:**
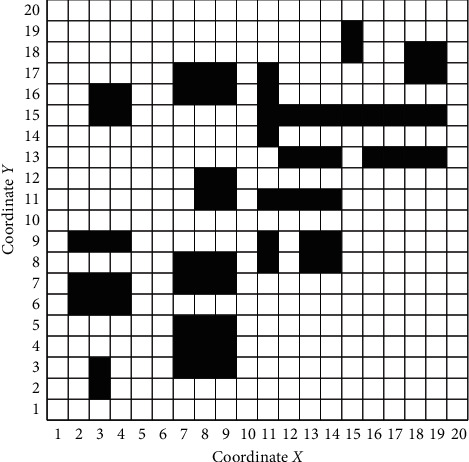
A 20 *∗* 20 grid simulation map.

In the 20 *∗* 20 grid map of Figure 5, the black obstacles represent infeasible areas, and the white grids represent feasible areas. The mobile robot enters at grid 0 and leaves from grid 399 (as shown in Figure 5).

#### 5.2.1. Algorithm Parameter Setting

For genetic algorithm, parameter setting is very important. It will directly affect the efficiency of the algorithm and the quality of path planning. The methods of selecting parameters usually include the empirical method, reference method, and exhaustive method. The empirical method means that we need to estimate a set of parameters based on our own experience. Obviously, this method is very unscientific. The reference method means that we need to refer to other literature studies on parameter setting. This method has certain rationality because the parameter setting in other literature studies has been verified and judged by the literature experiment, but this method has some limitations because each experimental environment has different requirements for parameters. Parameters that might have performed well in the previous experimental environment did not perform well in this experiment. The exhaustive method needs a large number of parameter combinations and experiments. This means that the exhaustive method needs a lot of time and computing resources to get a set of theoretically optimal parameters. Therefore, the exhaustive method is not practical.

In many literature studies, the control variable method is used to design the experimental parameters. They keep one variable changed and control the others unchanged. Then, the optimal value of this variable is obtained by experiment. They combined the optimal values of all the variables into a set of experimental parameters. This seems like a reasonable approach, but they ignore the fact that the relationship between variables is not independent, but interrelated. Taking the optimal value of each variable may lead to poor experimental results because variables are interrelated.

In [28], Chico Hermanu Brillianto Apribowo et al. used the DOE method to optimize the parameters of the genetic algorithm and achieved good experimental results. In [29], Mohsen Mosayebi and Sodhi not only optimized the parameters of the genetic algorithm using the DOE method but also guided the selection of genetic operators. In [30], Aldy Gunawan et al. presented a framework based on DOE to find a good initial range of parameter values for automated tuning. In [31], the correlation between the parameters and the sensitivity of the input parameters were revealed by Bettemir. Ö.H.

Based on [28–31], we consider using the DOE method to obtain a set of high-quality algorithmic parameters. Next, we will take AGACD algorithm as an example to introduce (experimental environment is a 20 *∗* 20 grid simulation map).

First, we need to determine which variables are sensitive to the algorithm. Then, the range of each sensitive variable is determined by the empirical method and reference method. We mark the lower limit of each variable as −1, the middle value as 0, and the upper limit as 1. The next step is to use the five-factor three-level combination table to represent the Taguchi table (Table 3).

The advantage of using the DOE method is that it is able to reduce the load of trying all possibilities' available solutions. It can also be used to find combinations of parameters for good results. The prediction equation can be approximated as follows:

(30) $$\begin{array}{c} \text{result}=X0+X1*F1+X2*F2+X3*F3+X4*F4+X5*F5. \end{array}$$

Based on Table 3, we can use the DOE method to determine the fixed variables of AGACD (Table 4).

Based on the algorithm parameters in Table 4, we performed 5 experiments for each group of parameters and then took the average value. The resulting data are shown in Table 5.

Based on Table 5, we can get the prediction equation. The prediction equation is as follows:

(31) $$\begin{array}{c} \text{result}=0.24416+X1*0.00673+X2*0.00847-X3*0.00841+X4*0.00594+X5*0.01624. \end{array}$$

Equation (31) will be maximal with *X*1 = 1, *X*2 = 1, *X*3 = −1, *X*4 = 1, and *X*5 = 1. The predicted value is 0.28995. Therefore, the high-quality parameter combination of AGACD is population = 60, pc_low = 0.65, pc_high = 0.7, pm_low = 0.15, and pm_high = 0.3. We used this combination of parameters to carry out 5 experiments. The data obtained are 0.3077, 0.2398, 0.2398, 0.4392, and 0.3077, respectively. The average value of the 5 data is 0.30684. The experimental results show that the actual value is better than the predicted value. So, we use the above parameter combination as the algorithm parameter of AGACD.

Similarly, we use the DOE method to obtain a high-quality parameter combination of BGA algorithm. This process is no longer described in detail. The high-quality parameter combination of BGA is population = 60, crossover probability = 0.8, and mutation probability = 0.3.

The design parameters for the two algorithms are shown in Table 6.

#### 5.2.2. Path Generation

**Figure 6:**
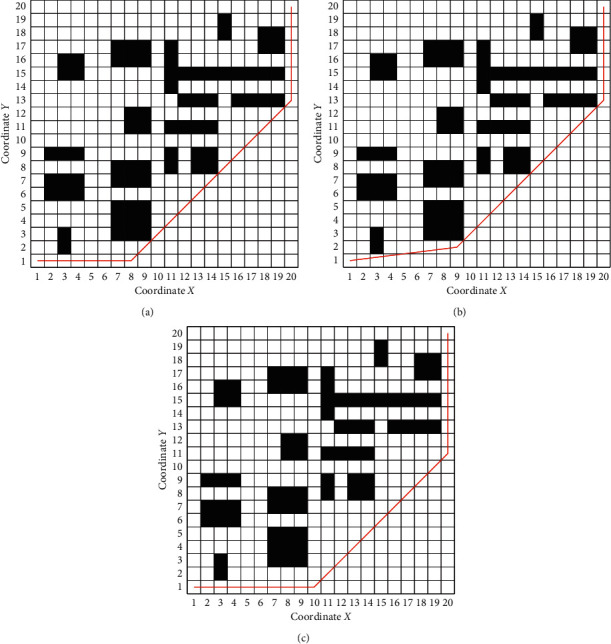
The experimental path of the two algorithms. (a) AGACD without the optimization operator. (b) AGACD with the optimization operator. (c) BGA.

The experimental paths of the two algorithms are shown in Figure 6.  Figure 6(a) shows the path generated by AGACD that does not use an optimization operator, and Figure 6(b) shows the path generated by AGACD that uses an optimization operator. By comparing the two figures, we can find that the path shown in Figure 6(a) can form the path shown in Figure 6(b) after optimization. The optimization operator is composed of the deleting point method and collision detection algorithm, and the use of the deleting point method is inseparable from the collision detection algorithm. Therefore, by comparing the path shown in Figure 6(a) with the path shown in Figure 6(b), the function and practicability of the collision detection algorithm can be fully demonstrated.  Figure 6(c) shows the path generated by BGA.

It can also be seen from Figure 6 that both algorithms can generate effective paths in the simulated map. From the quality of the generated path, the path generated by AGACD is superior to the path generated by BGA in path length and path smoothness. However, in terms of path safety, AGACD is slightly worse than BGA.

#### 5.2.3. Optimal Individual Fitness Analysis

**Figure 7:**
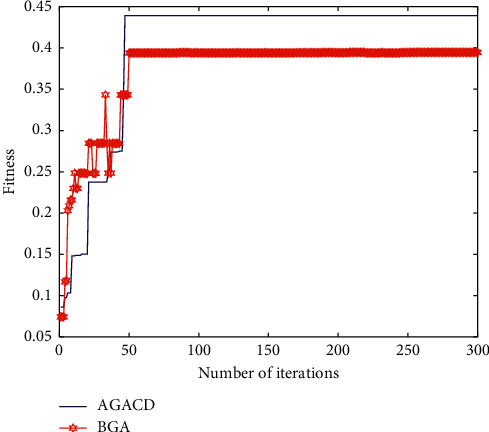
Evolutionary diagram of optimal individual fitness.

The evolution process of the optimal individual fitness of the two algorithms is shown in Figure 7. In terms of the number of convergence iterations, AGACD is better than BGA. This is because AGACD uses an adaptive crossover mutation operator and improves the traditional roulette selection strategy. These improvements can further accelerate the convergence speed of the algorithm. From the quality of convergent individuals, AGACD is better than BGA. This is because the optimization operator proposed by AGACD can make the second optimization of the convergent individuals and thus can improve the quality of the convergent individuals.

#### 5.2.4. Time Analysis

Table 7 details the program running time of each stage of AGACD and BGA during a program running process. Table 7 shows that the program running time of AGACD is less than the program running time of the BGA. The main reason is that the initial population generation time of AGACD is relatively short, and the proportion of the initial population generation time to the total time is relatively low. This shows that the improvement of the population initialization process in this paper is feasible and effective. By improving the population initialization process, the program running time of the algorithm is obviously reduced.

#### 5.2.5. Comprehensive Comparison

In the simulation environment of the 20 *∗* 20 grid map, each algorithm is simulated 20 times, and the average value is taken. We obtain the data shown in Table 8. Table 8 shows that AGACD is better than BGA in average fitness, average convergence iteration number, and average program running time.

The standard deviation is a measure of the dispersion of the mean of a set of data. A larger standard deviation indicates a larger difference between most values and their mean, and a smaller standard deviation indicates that these values are closer to the mean. So, we use the standard deviation to measure the average in Table 8. The standard deviation is shown in Table 9. As can be seen from Table 9, the value of AGACD is slightly larger than that of BGA in terms of standard deviation of average fitness. As a result, the data of BGA are closer to the average. The data of AGACD are relatively discrete. In the standard deviation of average convergence iteration number, the value of AGACD is much less than the value of BGA. So, the data of AGACD are much closer to the average and much less discrete. The data of BGA are very discrete. In the standard deviation of average program running time, the value of AGACD is much less than the value of BGA. So, the data of AGACD are much closer to the average and much less discrete. The data of BGA are very discrete.

In order to support these analyses and conclusions, we make appropriate statistical analysis on the simulation data of the algorithm.

**Figure 8:**
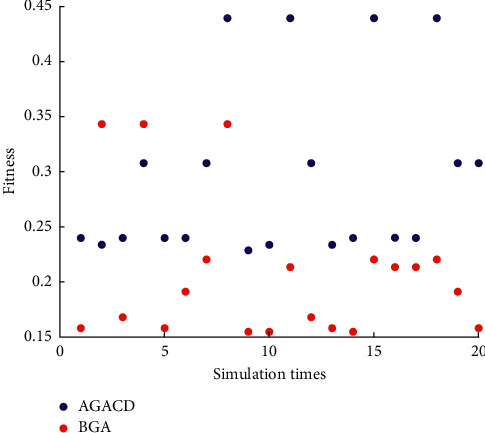
Scatter diagram of optimal individual fitness.

In Figure 8, the number of times AGACD outperforms BGA is 18. It accounts for 90% of all simulations. It means that, in most cases, AGACD produces better individuals than BGA. Furthermore, the optimal individual fitness of AGACD can reach 0.4392. By contrast, the best individual fitness produced by BGA is only 0.3431. The worst individuals produced by AGACD are also much better than those produced by BGA. Therefore, AGACD is superior to BGA in terms of optimal individual fitness.

**Figure 9:**
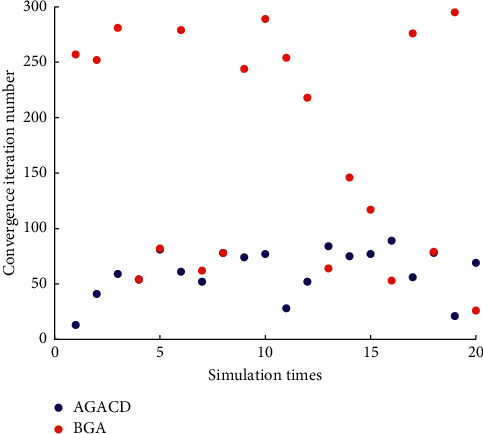
Scatter diagram of convergence iteration number.

As can be seen from Figure 9, the number of iterations required for BGA convergence is generally high. Convergence over 200 generations is 50% of the total number of times. The number of iterations required for AGACD to converge is about 60 generations. In almost every simulation, the number of iterations of AGACD convergence is less than that of BGA convergence. Therefore, AGACD is much better than BGA in terms of convergence iteration number.

**Figure 10:**
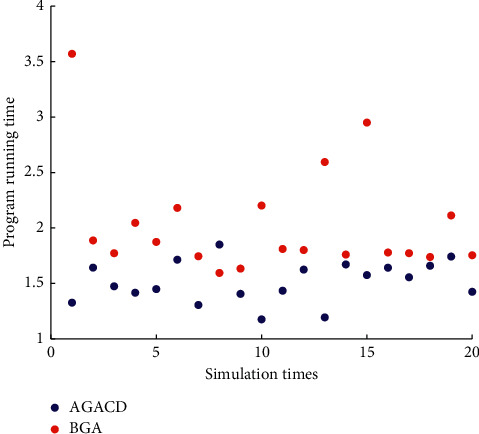
Scatter diagram of program running time.

In Figure 10, the number of times AGACD outperforms BGA is 19. It accounts for 95% of all simulations. This means that, in most cases, the AGACD program runs faster than the BGA program. And the running time of the AGACD program is usually concentrated in 1.5 seconds. The running time of the BGA program is usually concentrated in 2 seconds. Therefore, AGACD is superior to BGA in terms of program running time.

### 5.3. Simulation Experiment Based on the 50 *∗* 50 Grid Map

**Figure 11:**
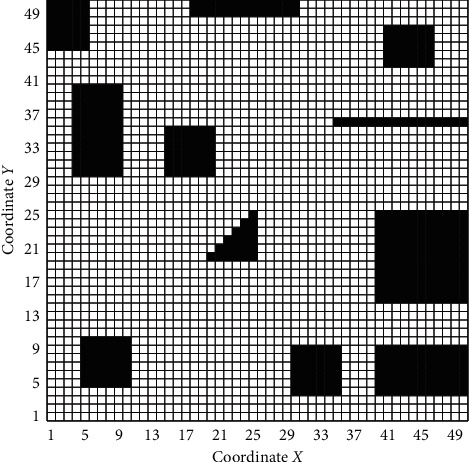
A 50 *∗* 50 grid simulation map.

In the 50 *∗* 50 grid map, the black obstacles represent infeasible areas, and the white grids represent feasible areas. The mobile robot enters from grid 0 and leaves from grid 2499 (as shown in Figure 11).

#### 5.3.1. Algorithm Parameter Setting

We will use the DOE method to obtain the high-quality parameter combination of the three algorithms. The specific process is described in Section 5.2.1. It will not be described in detail here. The design parameters for the three algorithms are shown in Table 10.

#### 5.3.2. Path Generation

**Figure 12:**
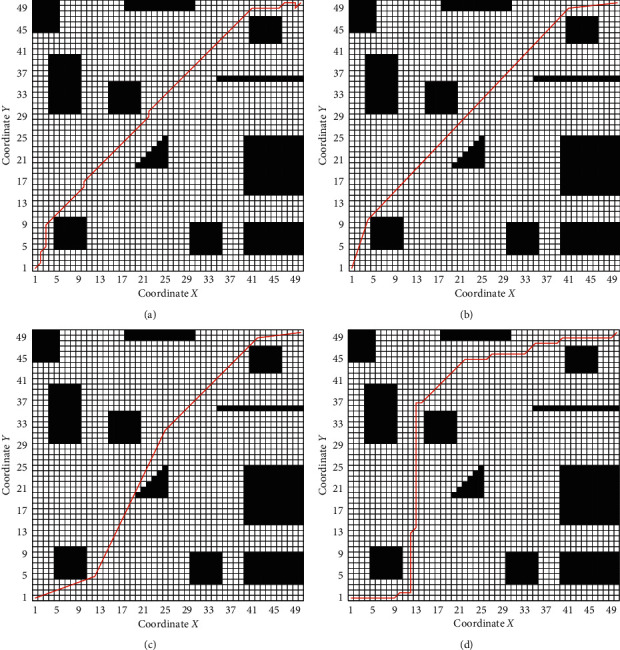
The experimental path of the three algorithms. (a) AGACD without the optimization operator. (b) AGACD with the optimization operator. (c) MPMGA. (d) BGA.

The experimental paths of the three algorithms are shown in Figure 12.  Figure 12(a) shows the path generated by AGACD that does not use an optimization operator, and Figure 12(b) shows the path generated by AGACD that uses an optimization operator. By comparing the two figures, we can find that the path shown in Figure 12(a) can form the path shown in Figure 12(b) after optimization. The optimization operator is composed of the deleting point method and collision detection algorithm used inseparably. By comparing the path shown in Figure 12(a) with the path shown in Figure 12(b), the function and practicability of the collision detection algorithm can be fully demonstrated.  Figure 12(c) shows the path generated by the MPMGA.  Figure 12(d) shows the path generated by BGA.

It can also be seen from Figure 12 that all algorithms can generate effective paths in the simulated map. With respect to path length and path smoothness, the path generated by AGACD is superior to the path generated by BGA and the path generated by MPMGA. However, in terms of path safety, AGACD is better than MPMGA but worse than BGA.

#### 5.3.3. Optimal Individual Fitness Analysis

**Figure 13:**
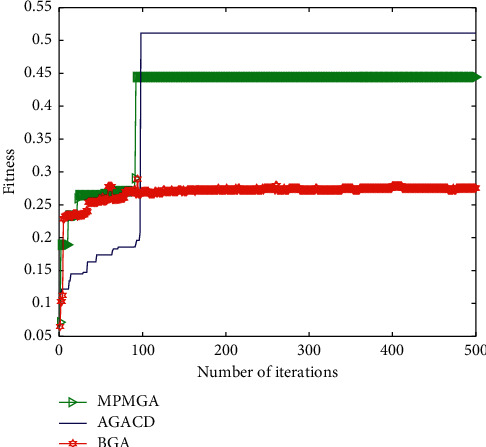
Evolutionary diagram of optimal individual fitness.

Figure 13 shows the evolutionary process of optimal individual fitness under current parameter settings. In terms of the number of convergence iterations, AGACD is better than BGA. This is because AGACD uses an adaptive crossover mutation operator and improves the traditional roulette selection strategy. These improvements can further accelerate the convergence speed of the algorithm. However, the number of convergence iterations of AGACD is almost the same as the number of convergence iterations of MPMGA. This shows that the convergence speeds of the two algorithms are almost the same. From the quality of convergent individuals, AGACD is better than BGA and MPMGA. This is because the optimization operator proposed by AGACD can perform a second optimization of the convergent individuals and thus can improve the quality of the convergent individuals.

#### 5.3.4. Time Analysis

Table 11 details the program running time of each stage of AGACD, BGA, and MPMGA during a program running process. By comparing Tables 7 and 11, we can find that, with increasing map size, the average program running time of BGA also increases sharply. The ratio of the initial population generation time to the total time of BGA increased from 15.6% to 82.34%. In the simulation environment of the 50 *∗* 50 grid map, the program running time of MPMGA reached an astonishing 62.6864 seconds. The time taken to generate the initial population accounted for 85.62% of the total time. However, the program running time of AGACD only increases slightly with the increased map size. The time taken to generate the initial population accounted for only 10.14% of the total time. Through the above comparative analysis, we believe that AGACD proposed in this paper can effectively reduce the running time of the program.

#### 5.3.5. Comprehensive Comparison

In the simulation environment of the 50 *∗* 50 grid map, each algorithm is simulated 20 times, and the average value is taken. We obtain the data shown in Table 12. Table 12 shows that AGACD is better than BGA and MPMGA in average fitness, average convergence iteration number, and average program running time. By comparing the average program running time of Tables 8 and 12, we can find that the average program running time of BGA increases sharply with the increased map size. In the simulation environment of the 50 *∗* 50 grid map, the average program running time of MPMGA reaches an astonishing 55.69 seconds. However, the average program running time of AGACD only increases slightly with the increased map size.

The standard deviation is a measure of the dispersion of the mean of a set of data. A larger standard deviation indicates a larger difference between most values and their mean, and a smaller standard deviation indicates that these values are closer to the mean. So, we use the standard deviation to measure the average in Table 12. The standard deviation is shown in Table 13. As can be seen from Table 13, the value of AGACD is larger than that of BGA in terms of standard deviation of average fitness, but the value of AGACD is smaller than that of MPMGA. As a result, the data of BGA are closer to the average. The data of AGACD are relatively discrete. In the standard deviation of average convergence iteration number, the value of AGACD is much less than the value of BGA and MPMGA. So, the data of AGACD are much closer to the average and much less discrete. The data of BGA and MPMGA are very discrete. In the standard deviation of average program running time, the value of AGACD is much less than the value of BGA and MPMGA. So, the data of AGACD are much closer to the average and much less discrete. The data of BGA and MPMGA are very discrete.

In order to support these analyses and conclusions, we make appropriate statistical analysis on the simulation data of the algorithm.

**Figure 14:**
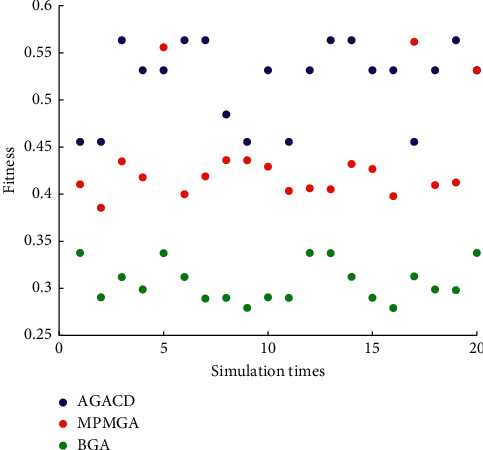
Scatter diagram of optimal individual fitness.

In Figure 14, the number of times AGACD outperforms BGA is 20. It accounts for 100% of all simulations. The number of times AGACD outperforms MPMGA is 17. It accounts for 85% of all simulations. It means that, in most cases, AGACD produces better individuals than BGA and MPMGA. Furthermore, the optimal individual fitness of AGACD can reach 0.5634. By contrast, the best individual fitness produced by BGA is only 0.3376. The worst individuals produced by AGACD are also much better than those produced by BGA. Therefore, AGACD is superior to BGA and MPMGA in terms of optimal individual fitness.

**Figure 15:**
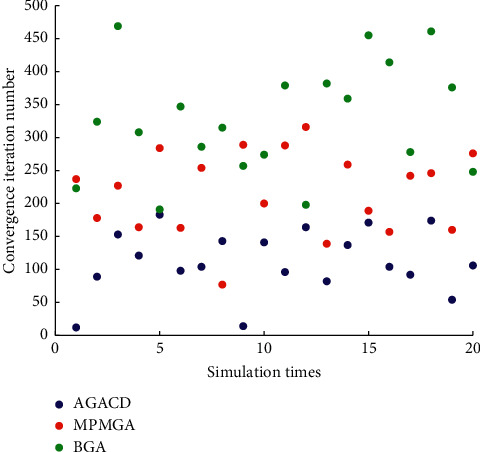
Scatter diagram of convergence iteration number.

As can be seen from Figure 15, the number of iterations required for BGA and MPMGA convergence is generally high. In almost every simulation, the number of iterations of AGACD convergence is less than that of BGA and MPMGA convergence. Therefore, AGACD is much better than BGA and MPMGA in terms of convergence iteration number.

**Figure 16:**
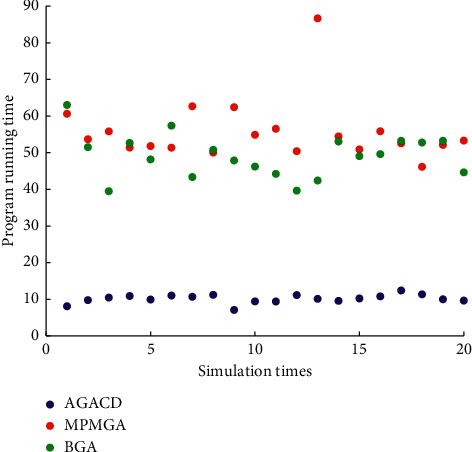
Scatter diagram of program running time.

In Figure 16, the number of times AGACD outperforms BGA is 20. It accounts for 100% of all simulations. The number of times AGACD outperforms MPMGA is 20. It accounts for 100% of all simulations. This means that, in most cases, the AGACD program runs faster than the BGA and MPMGA program. And the running time of the AGACD program is usually concentrated in 10 seconds. The running time of the BGA and MPMGA program is usually concentrated in 50 seconds. Therefore, AGACD is superior to BGA in terms of program running time.

Through the above simulation analysis, we can obtain the following conclusions:

1. Regardless of the simulation environment, AGACD is feasible and effective.
2. With the increase in the scale of the simulation map, the average program running time of BGA increases sharply. In the large-scale simulation map, the average program running time of MPMGA is even longer than the average program running time of BGA. The average running time of AGACD only increases slightly with increasing map size. Compared with other improved algorithms, AGACD can effectively reduce the running time of the program.
3. AGACD is superior to BGA and MPMGA in average fitness, average convergence iteration number, and average program running time.

## 6. Conclusions

In this paper, a path planning method of an adaptive genetic algorithm based on collision detection is proposed, and the operators of the basic genetic algorithm are improved. In terms of population initialization, this paper uses a priori knowledge and the random disturbance method. This population initialization method can effectively reduce the running time of the algorithm program and ensure the diversity of the initial population. In terms of fitness, this paper uses the Delphi weight method to evaluate the weight of each optimization objective to obtain a set of weights close to the actual situation. This fitness calculation method enhances the application ability of the algorithm in real life. In terms of the selection operator, this paper adopts the method of combining the roulette selection strategy with the elite selection strategy. On the one hand, this selection strategy can accelerate the convergence speed of the algorithm. On the other hand, this selection strategy can prevent the algorithm from data fallback phenomena. In terms of the crossover operator and mutation operator, an adaptive crossover operator and adaptive mutation operator are proposed in this paper. These two adaptive operators can not only protect high-quality individuals to speed up the convergence rate of the algorithm but also enhance the evolutionary potential of low-quality individuals to increase the searching ability of the algorithm. To improve the quality of convergent individuals, an optimization operator is proposed in this paper. The optimization operator can perform a second optimization on the convergent optimal individual. The proposed new operator or the improvement of the original operator can make the algorithm avoid the local optimal solution, accelerate the convergence speed of the algorithm, and improve the quality of the convergent individual. Simulation results show that the adaptive genetic algorithm based on collision detection is not only suitable for simulation maps of various sizes and obstacles but also has superior performance. In the same simulation map, compared with other improved algorithms, the program running time of the adaptive genetic algorithm based on collision detection is also greatly reduced.

## 7. Future Work

In this paper, the adaptive genetic algorithm based on collision detection is not only suitable for simulation maps of various sizes and obstacles but also has superior performance. However, the adaptive genetic algorithm based on collision detection also has obvious disadvantages. First, the mechanical property constraints of the robot are not considered, for example, the robot's minimum turning radius, maximum acceleration, maximum driving force, and maximum driving distance. Second, path planning in dynamic or unknown environments is not considered. The adaptive genetic algorithm based on collision detection proposed in this paper is suitable for global path planning in static environments. It does not take into account dynamic or unknown environments. Therefore, in real life, the application scenario of this algorithm is limited. In the future, we need to address three aspects. First, we need to add mechanical performance constraints to the algorithm. Second, we need to study and improve the algorithm to enhance its application ability in dynamic or unknown environments. Finally, we consider applying AGACD on actual mobile robots. Mobile robots can obtain information about obstacles in the surrounding environment through visual sensors to generate electronic maps (Castaño et al. [23, 24] introduced how to identify and detect obstacles under different weather conditions by using radar or sensor, so as to build an electronic map). We need to use the grid environment modeling method and map preprocessing process given in Section 2 to process the electronic map. After processing the electronic map, we get a grid map that algorithm can run. Then, we can run the AGACD program on the grid map to generate the actual path we need.

## Figures and Tables

**Table 1:** Importance ratio table.

Ratio *I*_*k*_	Description
1.0	The former is as important as the latter
1.2	The former is slightly more important than the latter
1.4	The former is more important than the latter
1.6	The former is much more important than the latter

**Table 2:** The hardware and software configuration.

Hardware	Processor	AMD Ryzen 5 4500U with Radeon Graphics 2.38 GHz
	RAM	8.00 GB (7.37 GB available)
Software	Operating system	Windows 10 (64 bit operating system)
	Simulation tool	MATLAB r2018a

**Table 3:** Testing level of fractional factorial DOE.

Population size			pc_low			pc_high			pm_low			pm_high		
Min	Mid	Max	Min	Mid	Max	Min	Mid	Max	Min	Mid	Max	Min	Mid	Max
40	50	60	0.55	0.6	0.65	0.7	0.75	0.8	0.05	0.1	0.15	0.2	0.25	0.3

Symbol of DOE			Symbol of DOE			Symbol of DOE			Symbol of DOE			Symbol of DOE		
−1	0	1	−1	0	1	−1	0	1	−1	0	1	−1	0	1

**Table 4:** Fixed variable of AGACD with the DOE method.

No.	Level factor					Real factor				
	Population	pc_low	pc_high	pm_low	pm_high	Population	pc_low	pc_high	pm_low	pm_high
1	40	0.55	0.7	0.05	0.25	−1	−1	−1	−1	0
2	40	0.6	0.75	0.1	0.3	−1	0	0	0	1
3	40	0.65	0.8	0.15	0.2	−1	1	1	1	−1
4	50	0.55	0.75	0.15	0.3	0	−1	0	1	1
5	50	0.6	0.8	0.05	0.2	0	0	1	−1	−1
6	50	0.65	0.7	0.1	0.25	0	1	−1	0	0
7	60	0.55	0.8	0.1	0.2	1	−1	1	0	−1
8	60	0.6	0.7	0.15	0.25	1	0	−1	1	0
9	60	0.65	0.75	0.05	0.3	1	1	0	−1	1

**Table 5:** Test data.

Trial 1	Trial 2	Trial 3	Trial 4	Trial 5	Average fitness	Factor (*X*)				
						*X*1	*X*2	*X*3	*X*4	*X*5
0.1386	0.2398	0.2551	0.2132	0.2398	0.2173	−0.2173	−0.2173	−0.2173	−0.2173	0
0.2398	0.2398	0.2336	0.2286	0.2169	0.2317	−0.2317	0	0	0	0.2317
0.2582	0.2337	0.2378	0.2337	0.2132	0.2353	−0.2353	0.2353	0.2353	0.2353	−0.2353
0.2398	0.2398	0.2336	0.2804	0.4392	0.2866	0	−0.2866	0	0.2866	0.2866
0.2398	0.2398	0.1364	0.2398	0.1364	0.1984	0	0	0.1984	−0.1984	−0.1984
0.2857	0.4392	0.2112	0.24	0.2398	0.2832	0	0.2832	−0.2832	0	0
0.2219	0.2857	0.1386	0.2398	0.2378	0.2248	0.2248	−0.2248	0.2248	0	−0.2248
0.2398	0.2378	0.2398	0.2114	0.2398	0.2337	0.2337	0	−0.2337	0.2337	0
0.2398	0.2398	0.2378	0.2453	0.4692	0.2864	0.2864	0.2864	0	−0.2864	0.2864
Average					0.24416	0.00673	0.00847	−0.00841	0.00594	0.01624

**Table 6:** Algorithm parameters.

AGACD	Start grid number	0	
	Target grid number	399	
	Initial population size	60	
	Number of iterations	300	
	Crossover probability	pc_high	0.7
		pc_low	0.65
	Mutation probability	pm_high	0.3
		pm_low	0.15

BGA	Start grid number	0	
	Target grid number	399	
	Initial population size	60	
	Number of iterations	300	
	Crossover probability	0.8	
	Mutation probability	0.3	

**Table 7:** Comparison table of the running time of each program.

Algorithm	Initial population generation time (s)	Population evolution time (s)	Total time (s)	Proportion of initial population generation time to total time
AGACD	0.0414	1.5886	1.63	2.54
BGA	0.3089	1.6711	1.98	15.60

**Table 8:** Data comparison table of the two algorithms.

Algorithm	Average fitness	Average convergence iteration number	Average program running time (s)
AGACD	0.2952	60.95	1.5125
BGA	0.2072	170.3	1.9508

**Table 9:** Standard deviation.

Algorithm	Standard deviation of average fitness	Standard deviation of average convergence iteration number	Standard deviation of average program running time
AGACD	0.0798	21.6004	0.1834
BGA	0.0639	100.788	0.4922

**Table 10:** Algorithm parameters.

AGACD	Start grid number	0
	Target grid number	2499
	Initial population size	80
	Number of iterations	500
	Crossover probability	pc_high	0.9
		pc_low	0.75
	Mutation probability	pm_high	0.3
		pm_low	0.15

BGA	Start grid number	0
	Target grid number	2499
	Initial population size	60
	Number of iterations	500
	Crossover probability	0.9
	Mutation probability	0.3

MPMGA	Start grid number	0
	Target grid number	2499
	Initial population size	45
	The size of each small population	15
	Number of iterations	500
	Crossover probability	High crossover probability	0.8
		Low crossover probability	0.3
	Mutation probability	High mutation probability	0.6
		Low mutation probability	0.1
	Accuracy coefficient	1
	Initial temperature	1
	Temperature decay rate	0.9

**Table 11:** Comparison table of the running time of each program.

Algorithm	Initial population generation time (s)	Population evolution time (s)	Total time (s)	Proportion of initial population generation time to total time (%)
AGACD	0.9777	8.664	9.6417	10.14
MPMGA	53.6721	9.0143	62.6864	85.62
BGA	43.3714	9.3021	52.6735	82.34

**Table 12:** Data comparison table of the three algorithms.

Algorithm	Average fitness	Average convergence iteration number	Average program running time (s)
AGACD	0.5197	111.9	10.16
MPMGA	0.4356	217.25	55.69
BGA	0.3065	327.2	49.13

**Table 13:** Standard deviation.

Algorithm	Standard deviation of average fitness	Standard deviation of average convergence iteration number	Standard deviation of average program running time
AGACD	0.0426	48.4886	1.1732
MPMGA	0.0514	62.3849	8.3968
BGA	0.0208	84.6433	5.9017

## Data Availability

The data used to support the findings of this study are included within the article. All required models and parameters are listed in the article.
